# Does a full-face helmet effectively protect against facial injuries?

**DOI:** 10.1186/s40621-019-0197-8

**Published:** 2019-06-01

**Authors:** Dan Wu, Marine Dufournet, Jean-Louis Martin

**Affiliations:** 1 Univ Lyon, Université Lyon 1, IFSTTAR, UMRESTTE UMR_T 9405, 25 Avenue François Mitterrand, 69675 Bron Cedex, France; 2 Clinical and Research Memory Centre of Lyon (CMRR); Geriatrics Unit, Lyon Institute For Elderly, Hospices civils de Lyon, Lyon, France

**Keywords:** MTW (motorized two-wheeler), Helmet type, Facial injury, Non-facial head injury, Head impact

## Abstract

**Background:**

The effectiveness of helmet use in preventing or reducing the severity of head injuries has been largely demonstrated. However, the effectiveness of different types of helmets in reducing facial or non-facial head injuries has received much less attention.

**Methods:**

A postal survey on motorized two-wheeler crashes was conducted in 2016. 7148 riders of motorized two-wheelers (MTW) injured in a crash between 2010 and 2014 and identified in the Rhône Trauma Registry were invited to complete a questionnaire in order to collect detailed information about their accidents. The analysis was based on a population of 405 helmeted riders who declared having received an impact on the head. Facial and non-facial head injury risks were estimated according to helmet type (full face or other) by logistic regression, controlled for type of object hit by the head (and gender for risk of non-facial head injury), and weighted to take nonresponse into account.

**Results:**

Three-quarter of helmeted MTW drivers were wearing a full-face helmet at the time of the accident. Victims wearing a full-face helmet were about three times less likely to have sustained injury to the face, compared to victims wearing another type of helmet (adjusted OR = 0.31; 95% CI: 0.11–0.83). On the other hand, the presence of non-facial head injury did not vary significantly according to whether a full-face or other helmet was worn (adjusted OR = 0.84; 95% CI: 0.33–2.13).

**Conclusions:**

Our study suggests that full-face helmets provide better facial protection for MTW users compared to other types of helmets, whereas there is no evidence of any difference in protection afforded the skull or the brain.

## Background

According to the International Transport Forum (ITF 2015), the number of road fatalities decreased by about 42% between 2000 and 2013 in the countries of the *International Road Traffic and Accident Database.* Mortality among light vehicle users declined by 54% in the same period, while the number of users of motorized two-wheelers (MTW) killed fell by only 22%. The result is an increase in the proportion of fatalities concerning MTW users relative to other road users. In France, they represented 21% of road deaths (18% for motorcyclists and 3% for moped riders) and 30% of those injured and hospitalized (21 and 9% respectively) in 2016 (ONISR 2017).

Many studies of the injury severity of crashes have examined the patterns and risk factors of MTW injuries as well as effective prevention programs for reducing them (Ankarath et al. 2002; Dischinger et al. 2006; Erhardt et al. 2016; Kraus et al. 1975; Liu et al. 2008; de Rome et al. 2012; de Rome et al. 2011). Lower-limb injuries are most common in all MTW crashes (Dischinger et al. 2006; Moskal et al. 2007; White et al. 2013) and head injuries are most frequent in fatal or serious crashes (Ankarath et al. 2002; Bachulis et al. 1988).

Wearing a helmet is an effective protection against head impact for MTW users and is nowadays obligatory in the great majority of countries. In France, mandatory helmet use was gradually introduced from 1961 to 1973 for motorcyclists and from 1976 to 1994 for moped riders (ONISR 2017). There are four major types of helmet: full-face, flip-up, open-face and half-cover. According to the national survey conducted in 2012 in France, 83% of MTW riders were equipped with a full-face or flip-up helmet and 19% equipped with an open-face helmet (SOeS 2013). Other types of helmet (mainly half-cover helmets) are not approved in France.

The effectiveness of helmet use in preventing or reducing the severity of head injuries was widely studied and demonstrated (Hurt et al. 1981; Khor et al. 2017; Liu et al. 2008; Moskal et al. 2008). However, the effectiveness of different types of helmets in reducing facial or non-facial head injuries received much less attention owing to the scarcity of information on helmet type in crash data. A few articles did study the association between helmet type and facial or non-facial head injuries (Cannell et al. 1982; Ramli et al. 2014; Ramli and Oxley 2016; Vaughan 1977; Yu et al. 2011). However, no article on this subject was found for France, apart from a study showing that helmeted moped riders suffered more facial injury than helmeted motorcyclists (13% compared to 8%), which, according to the authors, could be the consequence of better facial protection afforded by full-face helmets that are more often used by motorcyclists (Moskal et al. 2007).

The present study aims to estimate associations of motorcycle helmet type with occurrence of facial injury and non-facial head injury among riders of motorized two-wheelers who received an impact on the head, while controlling for potential confounders. We used a data-set which included helmet-type information, head-impact information and a comprehensive injury description, which are rarely simultaneously available.

## Materials and methods

This study relies on injury data from the Rhône Registry of Road Accident Victims coupled with data from a postal survey based on the Registry. The Registry collects medical data for all victims having a medical consultation following an injury road accident in the Rhône administrative Region of France (Laumon et al. 1997). From identification of the victims in the Registry, we were able to carry out a survey of accidents involving at least one MTW, with the aim of collecting detailed accident information.

### Data collection – survey

Between 2010 and 2014, 8022 MTW drivers aged 14 years and above, resident in France, and not killed in their accident, were identified by the Registry. After excluding 874 victims without valid home address, a letter was sent to 7148 victims to solicit their participation in the survey in 2016. They could reply by completing a paper questionnaire for return by post (in a prepaid envelope), or by completing an equivalent form online. Questions dealt with the circumstances of the accident, and the characteristics of the driver and the MTW. Details were sought about impacts received by MTW users, in particular impacts to the head, and protective equipment worn, notably the helmet (was a helmet worn, and if so, was it full-face or not).

### Injury definition

The outcomes of interest were facial injury and non-facial head injury. Injury data was provided from the Registry where every injury was coded according to the Abbreviated Injury Scale 1998 (AIS 98) (AAAM 1998). The AIS categorizes the facial region separately from the rest of the head (skull and brain). Facial injury was defined by the presence of any injury to the mouth, eyes, nose, ears or facial bones. Non-facial head injury was defined by the presence of any injury to the skull or brain.

### Case-control design

The case-control design was used to estimate the relative effect of two types of helmet (full-face or not) for facial injuries and non-facial head injuries. The first comparison to be made was between a group of victims who had received at least one facial injury (cases) and a group of victims who had sustained at least one injury elsewhere to the head (controls). The second comparison was between a group of victims who had received at least one non-facial head injury and the same control group as before. This control group was chosen because of the association between non-facial head and facial injuries reported in the literature (Hurt et al. 1981; Kraus et al. 2003). Kraus found that the risk of cranial trauma was 3.5 times higher when there was a facial wound and 6.5 times higher when there was facial fracture compared to users without any facial injury (Kraus et al. 2003).

### Potential risk factors

In the literature, some factors were considered confounding for the association between the type of helmet and injuries to the head: gender, age, alcohol consumption, driving speed, category of vehicle, type of accident, object hit by the head, etc. These factors were taken into account in analysis.

### Weighting for non-response

As with every survey soliciting volunteers, the response rate was low. However, the Registry provided information on non-respondents, reliably correcting the sample: by using probability models, it was possible to carry out an adjustment so that the respondents were representative of the source population (Brick 2013; Carlson and Williams 2001). As there are two causes of non-response to the survey, namely non-contact (incorrect address or no update since the accident) and refusal to reply, the adjustment for non-responses was carried out in two stages. The probability of contacting the subject (valid current address) was modeled by a first logistic regression, using the following factors: age, gender, residence in Rhône area, year of accident, accident site and type of road. The probability of the subject responding once contacted was established by a second logistic regression, using the following factors: age, gender, residence in Rhône area, type of MTW, helmet use, year of accident, accident site, type of road, third party involved and business trip. The corrective weighting for non-response was estimated as the inverse product of the two probabilities, and was applied to each estimate in the analyses.

### Missing data

Some factors such as the object hit by the head (9.9%) and the driving speed (7.7%) included missing values. For analytical simplicity, the missing values for object hit by the head were treated as an “unknown” category, and those for driving speed were imputed simply by linear regression, using auxiliary variables correlated to driving speed. Auxiliary variables used were: posted speed limit, type of road, urban/rural type of area, one-way road, intersection, road surface condition, accident configuration, driving situation with respect to speed (start, acceleration, braking, etc.), driving situation with respect to road (straight line, curve, turn, etc.), responsibility, high/inappropriate speed error, frequency of travel at the accident site, age, gender, driving license and season.

### Statistical analysis

Drivers wearing a full-face helmet and those wearing other types of helmet were compared in terms of the characteristics of the driver and vehicle, and the protective equipment worn, using the chi^2^ test. Univariate analyses were carried out to test the association between the occurrence of a facial injury or of a non-facial head injury and the type of helmet, as well as other factors listed above, by using logistic regression. Variables with *p*-value < 0.2 were selected for multivariate analysis, except for type of helmet, which was systematically included. All of the analyses were carried out using SAS version 9.4 (surveyfreq, surverylogistic procedures), taking into account corrective weightings for non-response.

## Results

Among the 7148 victims surveyed, 2475 never received the questionnaire as a result of obsolete postal addresses. These accounted for 35% of the non-contacts. Of the 4673 subjects with whom contact was established, 970 provided a usable questionnaire, yielding a final survey response rate of 21%. The response rate was higher for females, older riders, resident outside Rhône area, non-scooter riders, helmeted riders, and victims involved in an accident in 2014, outside Lyon, on a motorway/national road, with a third party, or during a home-work/business trip.

**Fig. 1:**
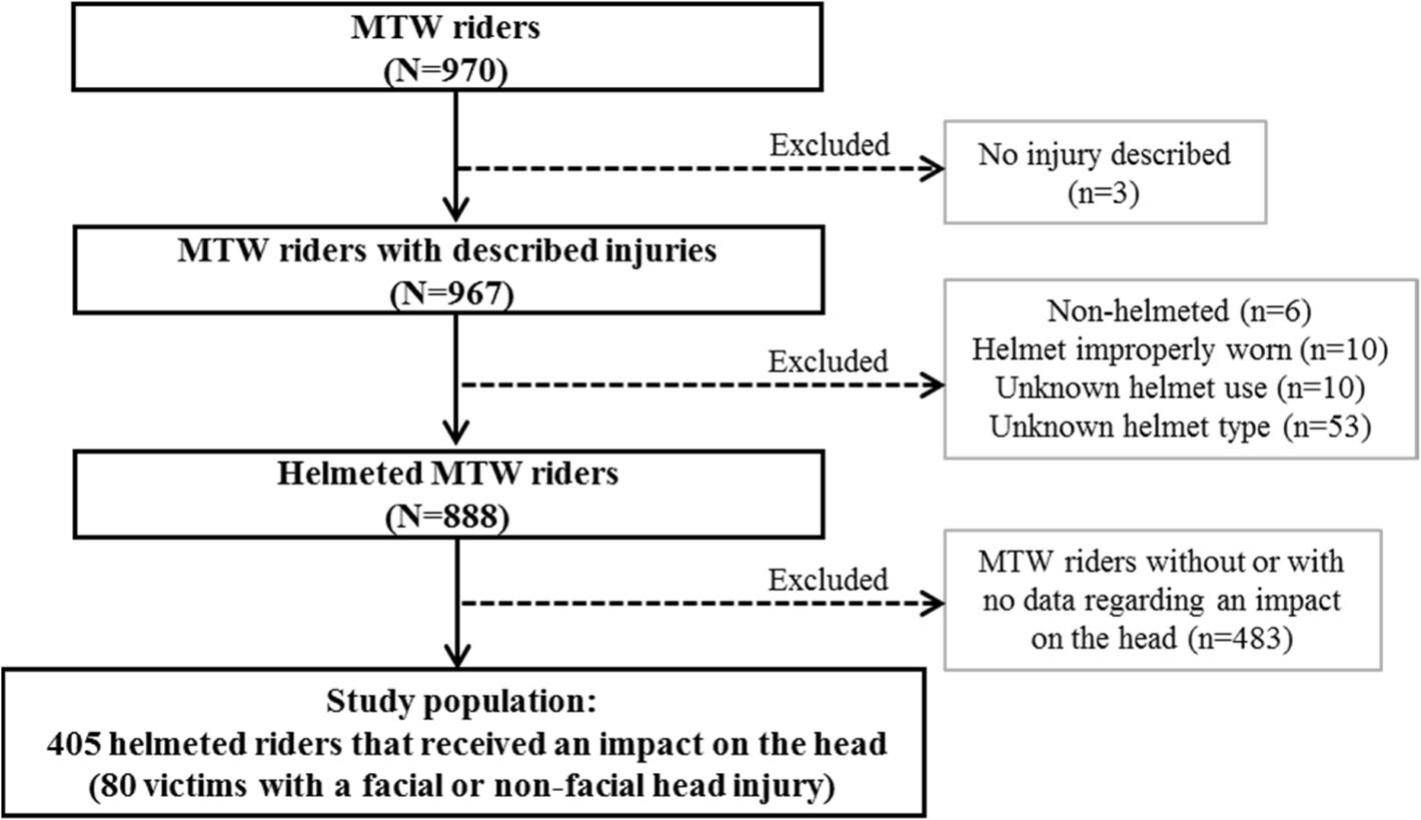
Study population selection flow-chart

Figure 1 describes how the study population was selected. Among the 970 usable responses, 3 were excluded due to the lack of injury description. Among others excluded, 6 victims were not wearing a helmet, 10 had a helmet that was badly fastened at the time of the accident, 10 had no information on whether a helmet was used, and 53 lacked information on helmet type. Among the 888 remaining victims, a little more than 45% (405) declared that they had received at least one head impact. Assuming that the effectiveness of the helmet could not be demonstrated without impact to the head, the study population was limited to only those victims wearing a correctly fastened helmet and having declared that they had sustained an impact to the head at the time of the accident. The study population therefore consisted of 405 victims (N_w_ = 3140, after weighting).

### Description of victims and profile of drivers wearing a full-face helmet

Among crash victims who suffered head impact, 9% were female and 48% were aged between 14 and 24 years (Table 1). For a large majority of victims, the crash occurred on a leisure trip (48%) or commute (38%). More than a third of victims were riding a moped (cylinder less than 50 cc). In terms of MAIS (Maximum Abbreviated Injury Scale), half of victims only sustained a minor injury (MAIS 1) and 15% at least a severe injury (MAIS ≥3).

**Table 1:** Description of victims according to type of helmet worn (full-face or other)

Characteristics	Population N_obs_ (%_w_)	Full-face N_obs_ (%_w_)	Other N_obs_ (%_w_)	*P*-value
Gender				0.190
Female	45 (8.5)	32 (7.4)	13 (12.0)	
Male	360 (91.5)	280 (92.6)	80 (88.0)	
Age				0.189
14–24	136 (47.7)	109 (47.8)	27 (47.3)	
25–49	179 (43.0)	142 (44.5)	37 (37.9)	
50+	90 (9.3)	61 (7.7)	29 (14.8)	
Alcohol consumption				0.012
No	398 (97.9)	311 (99.3)	87 (93.2)	
Yes	7 (2.1)	1 (0.7)	6 (6.8)	
Journey purpose				0.259
Home-work/school commute	182 (37.6)	149 (39.7)	33 (30.7)	
Work trip	19 (3.6)	11 (2.6)	8 (7.2)	
Leisure	163 (48.1)	123 (47.1)	40 (51.6)	
Other	41 (10.6)	29 (10.6)	12 (10.5)	
MTW category (cylinder capacity)				< 0.001
Moped (< 50 cm^3^)	102 (37.1)	69 (31.5)	33 (56.1)	
Light motorcycle (50–125 cm^3^)	67 (15.5)	42 (14.7)	25 (18.3)	
Heavy motorcycle (> 125 cm^3^)	236 (47.4)	201 (53.8)	35 (25.7)	
Motorcycle jacket				< 0.001
No	116 (36.6)	64 (27.3)	52 (68.5)	
Yes	289 (63.4)	248 (72.7)	41 (31.5)	
Motorcycle trousers				0.002
No	321 (80.0)	235 (75.9)	86 (93.9)	
Yes	84 (20.0)	77 (24.1)	7 (6.1)	
Knee-high boots, ankle boots				< 0.001
No	199 (57.5)	129 (49.9)	70 (83.6)	
Yes	206 (42.5)	183 (50.1)	23 (16.4)	
Back protection				< 0.001
No	279 (72.3)	194 (66.6)	85 (91.8)	
Yes	126 (27.7)	118 (33.4)	8 (8.2)	
Gloves				< 0.001
No	49 (17.0)	25 (9.7)	24 (41.8)	
Yes	356 (83.0)	287 (90.3)	69 (58.2)	
Retroreflective gear				0.003
No	340 (83.2)	254 (79.9)	86 (94.3)	
Yes	65 (16.8)	58 (20.1)	7 (5.7)	
Overall severity of injuries				0.610
MAIS 1	216 (54.4)	165 (54.2)	51 (55.1)	
MAIS 2	117 (30.4)	89 (29.4)	28 (33.7)	
MAIS 3+	72 (15.2)	58 (16.4)	14 (11.2)	
Head injury				0.007
No	325 (81.5)	260 (85)	65 (69.4)	
Yes	80 (18.5)	52 (15)	28 (30.6)	
Non-facial head injury				0.693
No	347 (86.9)	269 (87.3)	78 (85.4)	
Yes	58 (13.1)	43 (12.7)	15 (14.6)	
Facial injury				< 0.001
No	368 (91.2)	295 (95)	73 (78.2)	
Yes	37 (8.8)	17 (5)	20 (21.8)	

N_obs_ = number observed; %_w_ = weighted percentage

Eighty victims suffered from facial or non-facial head injuries (19%), 13 of them had serious head injuries (MAIS 3+), and 27 had a loss of consciousness. Non-facial head injury alone was present in 13% of victims and facial injury alone in 9%. Among facial injuries, the most frequent (53%) were abrasions, wounds and mucosal skin bruises. Simple bone injuries (39%) comprised bone fractures to the nose, jaw, eye socket, mandible or teeth. More complex ones were often located on the maxilla, sometimes leading to craniofacial separation.

Drivers wearing a full-face helmet are mostly drivers of large displacement motorbikes and are better equipped with protective clothing (jacket, trousers, gloves, boots and back protection) as well as retroreflective equipment. Drivers wearing other helmets are often users of MTWs with cylinders less than 50 cc.

### Protection given to the face and to the head excluding the face by wearing a full-face helmet

To evaluate the effectiveness of helmet use on facial and non-facial head injuries according to helmet type, two analyses were performed on these outcomes, respectively: occurrence of facial injury and occurrence of non-facial head injury. Table 2 shows the risk of facial injury in terms of the type of helmet, as well as other potential factors. The risk of facial injury was not associated with gender, age, cylinder capacity, type of accident or driving speed. It was associated, on the other hand, with the object hit during head impact, and the type of helmet worn. The risk of facial injury was much higher in case of collision with a vehicle or with a fixed object on the road or roadside, compared to a collision with the ground itself. Victims wearing a full-face helmet were at lower risk of sustaining facial injury, compared to those wearing other types of helmet, with an adjusted odds ratio (OR) of 0.31 (95% confidence interval (CI): 0.11–0.83).

**Table 2:** Risk of facial injury among victims with head impact

	Cases	Controls		
	%_w_ of victims with facial injury (N_obs_ = 37)	%_w_ of victims without head injury (N_obs_ = 325)	Crude OR (95% CI)	Adjusted OR (95% CI)
Type of helmet			*p* < 0.001	*p* = 0.020
Not full-face	56.0	19.3	Reference	Reference
Full-face	44.0	80.7	0.19 (0.08–0.44)	0.31 (0.11–0.83)
Object hit by the head			*p* < 0.001	*p* < 0.001
Ground/road	23.1	75.6	Reference	Reference
Vehicle	46.4	14.4	10.5 (3.49–31.65)	7.66 (2.38–24.65)
Fixed object	8.2	2.8	9.55 (1.95–46.85)	9.27 (2.14–40.07)
Unknown	22.3	7.2	10.1 (2.88–35.35)	7.29 (1.89–28.20)
Gender			*p* = 0.889	
Male	90.3	91.1	Reference	
Female	9.7	8.9	1.10 (0.30–3.99)	
Age			*p* = 0.720	
14–24	42.2	49.2	0.72 (0.29–1.76)	
25–49	49.7	41.5	Reference	
50+	8.1	9.3	0.73 (0.24–2.24)	
Alcohol consumption			*p* = 0.366	
No	95.6	98.0	Reference	
Yes	4.4	2.0	2.31 (0.37–14.33)	
MTW category (cylinder capacity)			*p* = 0.505	
Moped (< 50 cm^3^)	32.4	38.0	Reference	
Light motorcycle (50–125 cm^3^)	23.0	14.4	1.88 (0.58–6.14)	
Heavy motorcycle (> 125 cm^3^)	44.6	47.6	1.10 (0.41–2.98)	
Type of accident			*p* = 0.496	
Only MTW	26.7	33.2	Reference	
Other	73.3	66.8	1.37 (0.55–3.37)	
Driving speed of MTW	Mean of driving speed (km/h)		*p* = 0.729	
	47.7	49.0	1.00 (0.98–1.01)	

The risk of non-facial head injury was also studied with all the preceding factors taken into account. The corresponding results are shown in Table 3. Adjusted for gender and the object impacted by the head, the risk of suffering a non-facial head injury was not significantly different for victims wearing a full-face helmet, compared with those wearing other types of helmet, with an adjusted OR of 0.84 (95% CI: 0.33–2.13).

**Table 3:** Risk of non-facial head injury among victims with head impact

	Cases	Controls		
	%_w_ of victims with non-facial head injury (N_obs_ = 58)	%_w_ of victims without head injury (N_obs_ = 325)	Crude OR (95% CI)	Adjusted OR (95% CI)
Type of helmet			*p* = 0.408	*p* = 0.706
Not full-face	25.1	19.3	Reference	Reference
Full-face	74.9	80.7	0.71 (0.32–1.60)	0.84 (0.33–2.13)
Objet hit by the head			*p* = 0.161	*p* = 0.262
Ground/road	57.4	75.6	Reference	Reference
Vehicle	28.4	14.4	2.59 (1.06–6.32)	2.45 (0.95–6.30)
Fixed object	3.9	2.8	1.84 (0.43–7.90)	1.91 (0.44–8.27)
Unknown	10.3	7.2	1.88 (0.72–4.96)	1.71 (0.61–4.79)
Gender			*p* = 0.053	*p* = 0.073
Male	97.3	91.1	Reference	Reference
Female	2.7	8.9	0.28 (0.08–1.02)	0.30 (0.08–1.12)
Age			*p* = 0.779	
14–24	42.8	49.2	0.77 (0.36–1.66)	
25–49	46.9	41.5	Reference	
50+	10.3	9.3	0.98 (0.42–2.26)	
Alcohol consumption			*p* = 0.547	
No	99.0	98.0	Reference	
Yes	1.0	2.0	0.50 (0.05–4.81)	
MTW category (cylinder capacity)			*p* = 0.452	
Moped (< 50 cm^3^)	34.3	38.0	Reference	
Light motorcycle (50–125 cm^3^)	22.7	14.4	1.76 (0.62–4.99)	
Heavy motorcycle (> 125 cm^3^)	43.0	47.6	1.00 (0.44–2.27)	
Type of accident			*p* = 0.495	
Only MTW	27.4	33.2	Reference	
Other	72.6	66.8	1.32 (0.60–2.91)	
Driving speed of MTW*	Mean of driving speed (km/h)		*p* = 0.995	
	49.0	49.0	1.00 (0.98–1.01)	

Thus, on these two separate analyses, full-face helmets provided better facial protection, while type of helmet did not affect risks of skull or brain injury.

## Discussion

The present study investigated the added protection potentially provided by a full-face helmet, given that almost all users (98%) declared that they had worn a correctly fastened helmet. Information on the type of helmet collected through the survey enabled us to compare the relative effects of full-face and other types of helmet. However, for these “other” types of helmet, it was not known if the helmet was of the ‘modular’ type, in which case its effect may be very close to that of a full-face helmet, of the open-face type (with no chin strap), or of the half cover type (weak protection, not approved in France). In spite of this lack of precision, the present results were close to those of other previous studies.

### Comparison with other studies

There has been very little research into the relative effect of different types of helmet in protecting against facial or non-facial head injuries during MTW accidents. As regards facial injuries, three studies found that full-face helmets provided more protection than other types. For a full-face helmet, Vaughan showed that the risk of injury to the face was reduced by a half or two-thirds (Vaughan 1977); Brewer reported a 73% reduction in the relative risk of sustaining facial fracture (Brewer et al. 2013). Whitaker found that the rate of facial injury among wearers of full-face helmets was lower than for wearers of other types (7% vs. 24%) (Whitaker 1980). Two other studies suggested that full-face helmets afforded better protection than the ‘jets’ or other helmets (Cannell et al. 1982; Ramli et al. 2014). Our study showed that the full-face helmet lowered the risk of facial injury by two-thirds, and confirmed that a full-face helmet offers better protection against facial injury than other types of helmet.

As regards non-facial head injuries, some studies reported that other types of helmet were associated with a higher risk of non-facial head injuries than full-face helmets (Brewer et al. 2013; Tsai et al. 1995; Yu et al. 2011), while others studies did not find that the type of helmet made any difference in terms of non-facial head protection (Ramli et al. 2014; Vaughan 1977). Our study likewise did not reveal any difference in risks of non-facial head injuries according to helmet type.

### Strengths of the study

The main strong point of the study was that it used a sample including only MTW user accident victims who received a head impact during the accident. The data gave us access to rarely available information on the type of helmet, the type of object hit by the head, and the driving speed. For MTW users, direct impact to the head from an object is the most obvious cause of head injury (Cannell et al. 1982), and it is therefore very important to measure the effect of different types of helmet against facial or non-facial head injuries, adjusted to the type of object hit.

Using the Registry as a sample frame was an advantage for our survey. The Registry is an exhaustive data-base which identifies almost all road injury accident victims in the Rhône area. That allowed us to include mildly injured victims in the study population, which is not always the case in other studies (Brown et al. 2015; Cannell et al. 1982; Ramli et al. 2014).

### Limitations

The study had several limitations. Firstly, we identified three potential selection biases:

1. Having only data on injured victims at our disposal, the control group comprised helmeted victims with head impact but no head injury, but who sustained injury in at least one other body region. However, the ideal control group would have comprised helmeted victims who received a head impact without sustaining head injury, regardless of whether or not they received other injuries. In other words, helmeted victims who received an impact to the head but did not receive any injury are missing from the control group. Given that there is no reason why our group of specifically injured controls should be representative of both injured and non-injured MTW users with head impact, it is possible that there is a selection bias in the given estimates. Recent theoretical results, using the Structural Causal Model (Bareinboim et al. 2014; Bareinboim and Pearl 2012), formally demonstrated the existence of bias when selection depends on both exposure and the event of interest, both of which applied in the present study. On the one hand, selection based on injury in general depends on the protective equipment used and therefore on the type of helmet, while, on the other hand, the fact of being injured is not independent of head injury. It is interesting to reflect upon the meaning and magnitude of this proven bias, as other authors have done in road safety studies concerning bias in analyses of responsibility (Dufournet 2018; Dufournet et al. 2016). In the present case, if it is assumed that injury accident victims are not injured partly thanks to wearing better equipment, the rate of full-face helmet use was underestimated in the present control group, which in turn leads to an underestimation of the protective effect of the full-face helmet estimated by the present study.
2. Selection bias could exist due to survey non-respondents. The questionnaire response rate was relatively low, and the population of respondents differed from the non-respondent population, which could bias the results. The use of corrective weighting for non-response allowed us to minimize such possible bias, but this weighting could only take account of variables available in the Registry.
3. Selection bias could also exist due to several study sample selection factors described in Fig. 1: injury description, helmet type and head impact. The potential bias caused by the exclusion of three victims without injury data was negligible. However, excluding victims from the study due to missing data on helmet type and head impact could have an influence on the results of the study. Without knowing the reason for the missing data on these two factors, it is difficult to know what influence they might have on the results.

Secondly, we identified some information biases in the data collected by questionnaire.

1. Victims were asked to participate in the survey (between 2 and 7 years after the accident). This length of time that elapsed since the accident may have led to imprecise responses.
2. It is possible that some participants did not fully understand certain questions or gave an inexact response to questions liable to suggest responsibility for the accident (speed, alcohol consumption). In particular, the declared driving speed could often have been underestimated by victims. We used this driving speed as a proxy of the speed of head impact, and no effect on the occurrence of facial injuries or non-facial head injuries has been found, which might be due to underestimation of the speed reported by victims.
3. An important piece of information, the precise area of head impact, was not available. Consequently, the area of impact could not be considered in the study. It was known whether the driver’s head collided with an object and what kind of object was hit, but the area of impact on the driver’s helmet was not known. Therefore, when the impact did not involve the facial region, the estimated protective effect of the full-face helmet compared to other types of helmet against facial injuries could be biased.

## Conclusion

Our study suggests that the full-face helmet provide better facial protection than other types of helmet, while no difference is shown for skull and brain protection according to type of helmet. Facial injury can have esthetic, functional, sensory and psychological consequences, and our results clearly encourage MTW users to wear a full-face helmet.

## Abbreviations

AAAM: Association for the Advancement of Automotive Medicine
MTW: Motorized Two-Wheelers
